# Characterization and diagnostic application of genomic *NPM-ALK* fusion sequences in anaplastic large-cell lymphoma

**DOI:** 10.18632/oncotarget.25489

**Published:** 2018-05-29

**Authors:** Manuela Krumbholz, Wilhelm Woessmann, Jakob Zierk, David Seniuk, Paolo Ceppi, Martin Zimmermann, Vijay Kumar Singh, Markus Metzler, Christine Damm-Welk

**Affiliations:** ^1^ University Hospital Erlangen, Department of Pediatrics, Erlangen, Germany; ^2^ Justus-Liebig University, Department of Pediatric Hematology and Oncology, Giessen, Germany; ^3^ Junior Research Group 1, Interdisciplinary Centre for Clinical Research, Friedrich-Alexander-University Erlangen-Nürnberg (FAU), Erlangen, Germany; ^4^ Hannover Medical School, Department of Pediatric Hematology and Oncology, Hannover, Germany

**Keywords:** pediatric oncology, ALK-positive anaplastic large cell lymphoma, NPM-ALK fusion, genomic fusion sequences, minimal disease monitoring

## Abstract

*Nucleophosmin-anaplastic lymphoma kinase* (*NPM-ALK)* fusion genes resulting from the translocation t(2;5)(p23;q35) are present in almost 90% of childhood ALK-positive anaplastic large-cell lymphomas (ALCL). Detection and quantification of minimal disseminated disease (MDD) by measuring *NPM-ALK* fusion transcript levels in the blood provide independent prognostic parameters. Characterization of the genomic breakpoints provides insights into the pathogenesis of the translocation and allows for DNA-based minimal disease monitoring.

We designed a nested multiplex PCR assay for identification and characterization of genomic *NPM-ALK* fusion sequences in 45 pediatric ALCL-patients, and used the sequences for quantitative MDD monitoring. Breakpoint analysis indicates the involvement of inaccurate non-homologous end joining repair mechanisms in the formation of *NPM-ALK* fusions. Parallel quantification of RNA and DNA levels in the cellular fraction of 45 blood samples from eight patients with NPM-ALK-positive ALCL correlated, as did cell-free circulating *NPM-ALK* DNA copies in the plasma fraction of 37 blood samples. With genomic *NPM-ALK* fusion sequence quantification, plasma samples of ALCL patients become an additional source for MRD-assessment. Parallel quantification of *NPM-ALK* transcripts and fusion genes in ALCL cell lines treated with the ALK kinase inhibitor crizotinib illustrates the potential value of supplementary DNA-based quantification in particular clinical settings.

## INTRODUCTION

ALK-positive anaplastic large-cell lymphoma (ALCL) accounts for 10-15% of pediatric non-Hodgkin lymphoma (NHL) cases [1]. Almost 90% of pediatric ALCL carry the chromosomal translocation t(2;5)(p23;q35) resulting in the fusion of the *nucleophosmin* (*NPM*) gene and the *anaplastic lymphoma kinase* (*ALK*) gene [2–4]. The NPM-ALK fusion protein is a constitutively active tyrosine kinase that is heavily involved in tumor pathogenesis and maintenance [5].

The pathogenic mechanisms involved in the generation of the *NPM-ALK* fusion gene have not been analyzed in patients thus far. A systematic analysis of genomic fusion sequences from ALCL patients could provide insights into the pathogenesis of the translocation. The genomic fusion sites consistently fall within specific breakpoint cluster regions that comprise a 1 kb region around intron 4 within the *NPM* gene and a 2.2 kb region between exon 19 and exon 20 within the *ALK* gene [6, 7].

Standard multi-agent chemotherapy reaches event-free survival rates of 70% at five years [8–11]. New therapeutic options are available to be tested for patients with a high relapse risk in addition to chemotherapy (*e.g*., ALK-kinase inhibitors or Brentuximab Vedotin) or for those with a low relapse risk as a lower toxicity backbone (Vinblastine monotherapy) [12–14]. Therefore, reliable prognostic factors are necessary. The tumor-specific *NPM-ALK* fusion transcript has been established as a minimal disease marker in both bone marrow and blood mononuclear cells. Several groups have established detection protocols for minimal disseminated disease (MDD) by qualitative PCR for *NPM-ALK* mRNA as an independent and potent prognostic parameter under BFM pulse-type chemotherapy [15–19]. Fifty-five to sixty percent of patients are MDD-positive, and their risk of relapse is about 50% compared to 15% for MDD-negative patients [15–18]. Quantification of MDD has been shown by one group to detect patients with very high risk of relapse of 70% [17]. Detection of minimal residual disease (MRD) before the second course of chemotherapy allowed for definition of very high-risk patients with a relapse risk of almost 80%, as well [18]. However, despite the proven reliability of the MDD-marker at the RNA level, the use of RNA has some intrinsic disadvantages such as possible degradation by RNases during transport of blood samples to central laboratories. In addition, supplementary quantification of DNA fusion sequences would allow for calculation of absolute tumor cell numbers independent of gene expression, and detection of quiescent tumor cells. The fact that the breakpoint cluster regions in the *NPM* and *ALK* genes in ALCL are relatively small facilitates the design of fusion gene detection assays.

In the present study, we developed a nested multiplex PCR assay for identification of genomic *NPM-ALK* fusion sequences and performed a detailed characterization of the genomic breakpoints in pediatric ALCL. We evaluated the genomic fusion sequence as a supplementary tool for minimal disease assessment in both the cellular and plasma fractions of blood in children and adolescents with ALK-positive ALCL.

## RESULTS

### Characterization of genomic *NPM* and *ALK* breakpoints in ALCL patients

The nested multiplex PCR assay enabled identification of the genomic *ALK* fusion sequences in all four tested ALK+ cell lines (Karpas 299, SR-786, L-82, and SuDHL-1) and in all 45 ALCL patients (Table 1).

**Table 1:** Patient՛s characteristics and genomic breakpoint positions

Patient ID	Age at diagnosis (years)	Sex	Minimal disseminated disease	Break position *NPM1-ALK (GRCh37/hg19)*		Filler (bp)	Micro-homologies (bp)	Break position *ALK-NPM1 (GRCh37/hg19)*		gains and losses (bp)	
				*NPM1* (chr5:)	*ALK* (chr2:)			*ALK* (chr2:)	*NPM1* (chr5:)	*NPM1*	*ALK*
UPN1	7.2	f	negative	170,818,898	29,447,090	8	0	29,447,105	170,818,917	-18	-14
UPN2	14.8	m	positive	170,819,445	29,446,546	0	0				
UPN3	8.4	m	positive	170,819,197	29,447,296	0	2				
UPN4	15.7	m	positive	170,819,221	29,447,309	0	2				
UPN5	15.4	m	positive	170,818,960	29,447,677	0	0	29,447,718	170,819,094	-133	-40
UPN6	16.1	f	positive	170,819,226	29,447,374	0	0	29,447,408	170,819,307	-80	-33
UPN7	11.5	m	n.a.	170,819,006	29,448,395	0	2	29,448,420	170,819,032	-25	-24
UPN8	12	f	positive	170,819,682	29,447,119	0	0	29,447,177	170,819,740	-57	-57
UPN9	15	m	positive	170,819,566	29,447,140	0	0	29,447,199	170,819,643	-76	-58
UPN10	16.7	f	positive	170,818,883	29,448,215	0	1				
UPN11	14.4	m	positive	170,819,131	29,447,024	0	0	29,447,084	170,819,157	-23	-59
UPN12	10.1	m	negative	170,819,664	29,448,341	0	1	29,448,366	170,819,728	-63	-24
UPN13	7.2	m	negative	170,819,274	29,447,701	0	0	29,447,725	170,819,344	-69	-23
UPN14	3.9	f	positive	170,819,591	29,446,607	0	3	29,446,646	170,819,634	-42	-38
UPN15	4.6	m	n.a.	170,819,293	29,448,101	0	2				
UPN16	17.2	f	positive	170,819,070	29,447,988	0	0	29,448,046	170,819,099	-28	-57
UPN17	7.7	m	negative	170,819,660	29,447,251	0	0	29,447,282	170,819,686	-25	-30
UPN18	15.6	f	positive	170,818,991	29,448,172	0	1	29,448,178	170,819,017	-25	-5
UPN19	11.7	f	negative	170,819,466	29,447,635	0	3	29,447,657	170,819,527	-60	-21
UPN20	11.2	f	negative	170,819,157	29,447,876	0	0				
UPN21	15	f	positive	170,819,544	29,446,623	1	0	29,446,648	170,819,645	-100	-24
UPN22	13.8	f	positive	170,819,488	29,446,635	0	2	29,446,709	170,819,515	-26	-73
UPN23	8.1	m	negative	170,818,958	29,447,699	2	0	29,447,817	170,819,068	-109	-117
UPN24	3.5	f	negative	170,819,590	29,446,702	2	0				
UPN25	8.1	m	n.a.	chr2:216,195,742 **(ATIC)**	29,446,788	0	2				
UPN26	5.3	m	negative	170,819,658	29,448,164	0	1	29,448,178	170,819,732	-73	-13
UPN27	11.4	f	positive	170,819,690	29,448,219	2	0	29,448,355	170,819,742	-51	-135
UPN28	8.7	m	n.a.	chr1:154,130,428 **(TPM3)**	29,448,416	0	0				
UPN29	13.7	m	positive	170,819,465	29,447,764	1	0	29,447,878	170,819,504	-38	-113
UPN30	15.3	m	positive	170,819,429	29,447,734	1	0	29,447,806	170,819,515	-85	-71
UPN31	7.9	m	positive			0	0	29,448,297	170,818,878		
UPN32	8.7	m	positive	170,819,385	29,447,917	0	2	29,448,006	170,819,481	-95	-88
UPN33	12.7	f	positive	170,819,254	29,446,584	6	0				
UPN34	16.9	f	n.a.	170,819,500	29,447,765	0	0				
UPN35	13.3	f	n.a.	170,819,320	29,446,715	1	0	29,446,736	170,819,362	-41	-20
UPN36	2.5	f	n.a.	170,819,658	29,447,102	0	6	29,447,135	170,819,768	-109	-29
UPN37	14.4	m	positive	170,819,079	29,447,311	0	0	29,447,381	170,819,113	-33	-69
UPN38	3.1	f	negative	170,819,367	29,447,597	0	0				
UPN39	10.4	m	positive	170,819,176	29,447,498	0	0	29,447,548	170,819,238	-61	-49
UPN40	14.6	m	positive	170,819,186	29,447,815	0	0				
UPN41	4.2	f	negative	170,819,308	29,447,888	0	1	29,447,940	170,819,364	-55	-51
UPN42	7.4	m	negative	170,819,252	29,446,761	6	0	29,446,893	170,819,280	-27	-131
UPN43	12.2	m	positive	170,819,441	29,447,645	0	3	29,447,748	170,819,552	-110	-102
UPN44	5.6	m	positive	170,818,895	29,447,740	0	0	29,447,766	170,818,950	-54	-25
UPN45	14.9	m	positive	170,819,724	29,446,564	0	1				
Cell lines:											
Karpas 299	25	m	/	170,819,509	29,448,184	0	0				
SuDHL-1	10	m	/	170,819,668	29,447,024	7	0	29,447,105	170,819,618	51	-80
SR-786	11	m	/	170,819,199	29,446,897	0	0				
L-82	24	w	/	170,819,583	29,447,947	0	2				

n.a. not available.

**Figure 1:**
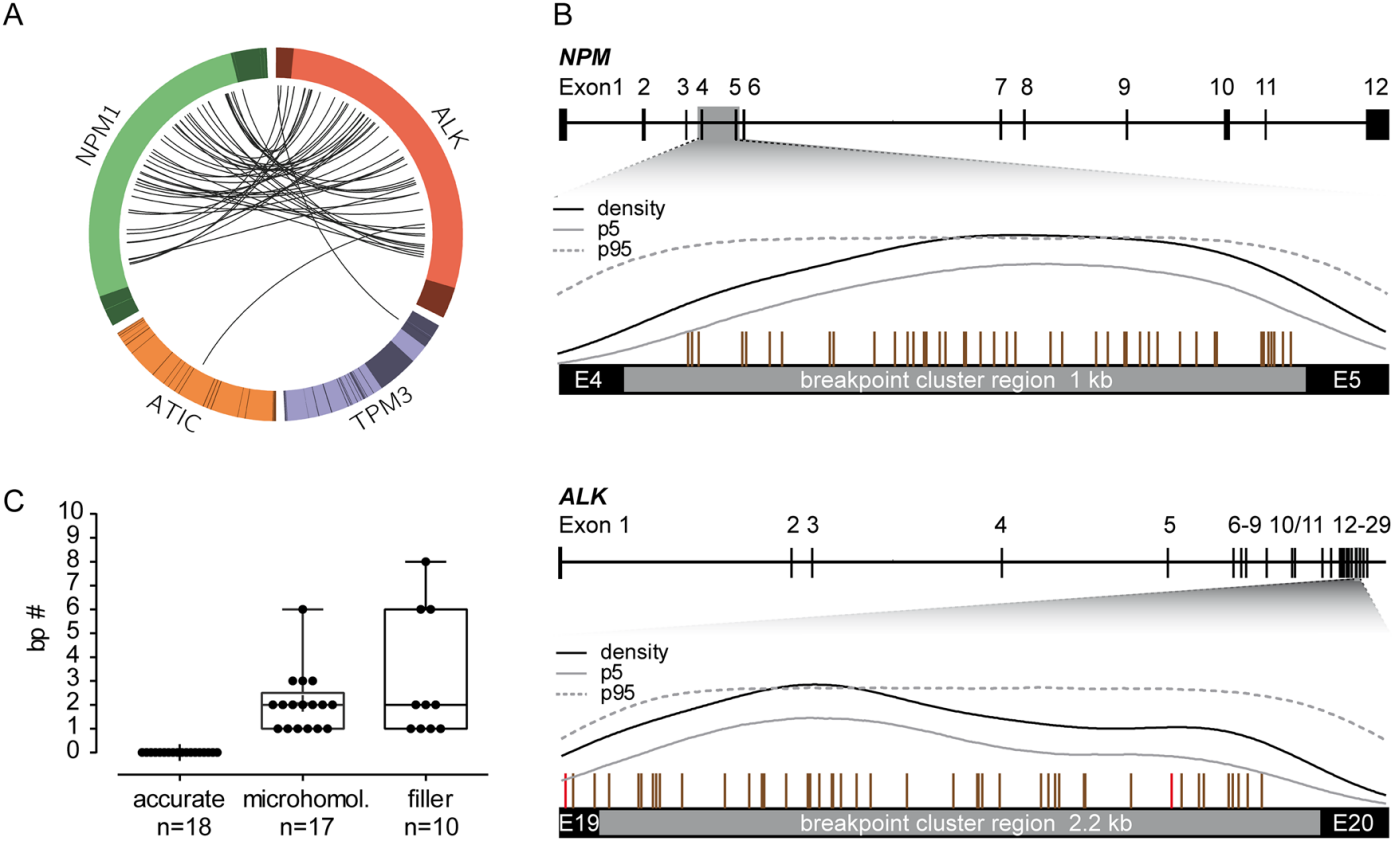
Breakpoint distribution in *ALK* and the respective fusion partner gene in 45 pediatric ALK-positive ALCL patients **(A)** Circos plot presents the genomic rearrangements within the *ALK* breakpoint cluster region (bcr), *NPM* bcr, *ATIC* gene, and *TMP3* gene. Exons are illustrated in darker colors. **(B)** Genomic organization of the *NPM* and *ALK* gene with the corresponding bcr. Vertical bars above the bcr represent individual genomic breakpoints. Results of Kernel density analysis: dashed line = breakpoint density; gray line = lower limit of 95% confidence band determined by bootstrapping procedure; black line = 95% confidence interval of a density function resulting from simulations at randomly distributed pseudo-breakpoints. **(C)** Boxplot represents the median and range of nucleotide numbers involved in microhomologies and fillers at the individual *NPM-ALK* fusion site.

In 43 patients, *NPM* was the fusion partner of *ALK*. *ATIC* and *TPM3* were the fusion partners in the two remaining patients, respectively (Figure 1A). In one patient (UPN31), the *NPM-ALK* fusion gene was not detectable, but the reciprocal *ALK-NPM* fusion gene could be sequenced. In 30 ALCL patients and one cell line (SuDHL-1), we were able to detect both derivative fusion sites (*NPM-ALK* and *ALK-NPM*). There were no cases with a perfectly balanced translocation: nearly all patients had deletions at the fusion region with a median deletion size of 55 base pairs (bp) in *NPM* and 49 bp in *ALK* (Table 1).

The alignment of the genomic breakpoints to the breakpoint cluster region (bcr) of *NPM* showed a random distribution with no sub-clusters (Figure 1A-1B). All *NPM* breakpoints identified were located in intron 4 and were randomly distributed therein. Genomic breakpoints within the *ALK* bcr were mostly located in intron 19 (93%), with 3 breakpoints in exon 19 (7%). Although genomic *ALK* breakpoints appeared to be enriched in the first half of intron 19, kernel density analysis did not identify any significant clustering (Figure 1B).

Detailed characterization of the *NPM-ALK* fusion sites showed small microhomologies (1 to 6 bp) in 38% of patients and small fillers (1 to 8 bp) in 22% of patients (Figure 1C). These findings indicate that the formation of *NPM-ALK* translocations in ALCL involves inaccurate non-homologous end joining (NHEJ) repair mechanisms [20].

**Figure 2:**
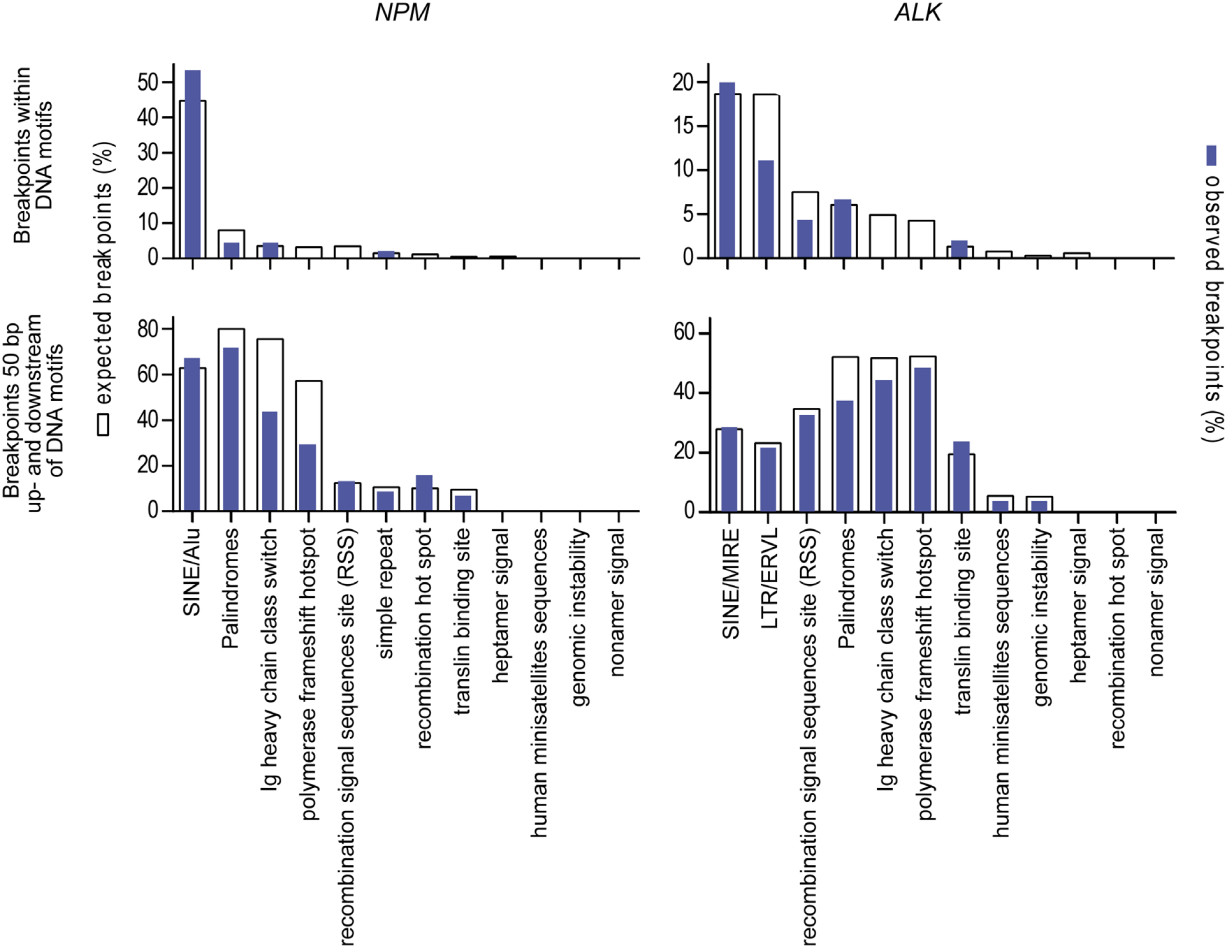
Localization of genomic breakpoints Co-localization of genomic breakpoints to repeat regions and DNA sequence motifs that could support the initiation of ALCL chromosomal translocation. White columns represent the numbers of expected breakpoints; blue columns represent the numbers of observed breakpoints within the corresponding DNA motif.

We further analyzed the genomic breakpoints for co-localization with repeat regions or with other DNA sequence motifs that could support the initiation of ALCL chromosomal translocation as described for other lymphoma subtypes [21, 22]. No significant correlation could be observed with repeat elements or other sequence motifs at the exact breakpoint position or expanded breakpoint regions (plus 50 bp upstream and downstream) that suggest an inaccurate DNA strand repair at the fusion site (Figure 2).

### Comparative quantification of *NPM-ALK* fusion transcripts and *NPM-ALK* fusion gene sequences in blood and plasma samples

To evaluate the potential application of DNA-based minimal disease monitoring for ALCL patients, we compared the standard RNA-based technique with DNA-based quantification using patients’ individual fusion sequences from both the cellular and plasma fractions in 51 blood samples. The 51 samples were collected from eight high-risk patients identified as MDD-positive by the standard method during the course of their treatment. Seven of the patients relapsed. Consequently, more than half the samples showed quantifiable copy numbers using RNA-based MDD/MRD measurement. This provided sufficient samples with measurable copy numbers for a quantitative comparison of the two methods.

*NPM-ALK* transcripts in the mononuclear cell fraction were quantifiable in 48 of the 51 samples (when applying the quality criteria of 2000 copies *ABL*). Of those 48 samples, 23 were negative and 25 positive. Three of the 48 samples quantifiable by RT-qPCR were not quantifiable by the DNA-based assay. Conversely, the DNA-based assay was able to quantify eight samples that could not be evaluated at the RNA-level (Supplementary Table 1).

**Figure 3:**
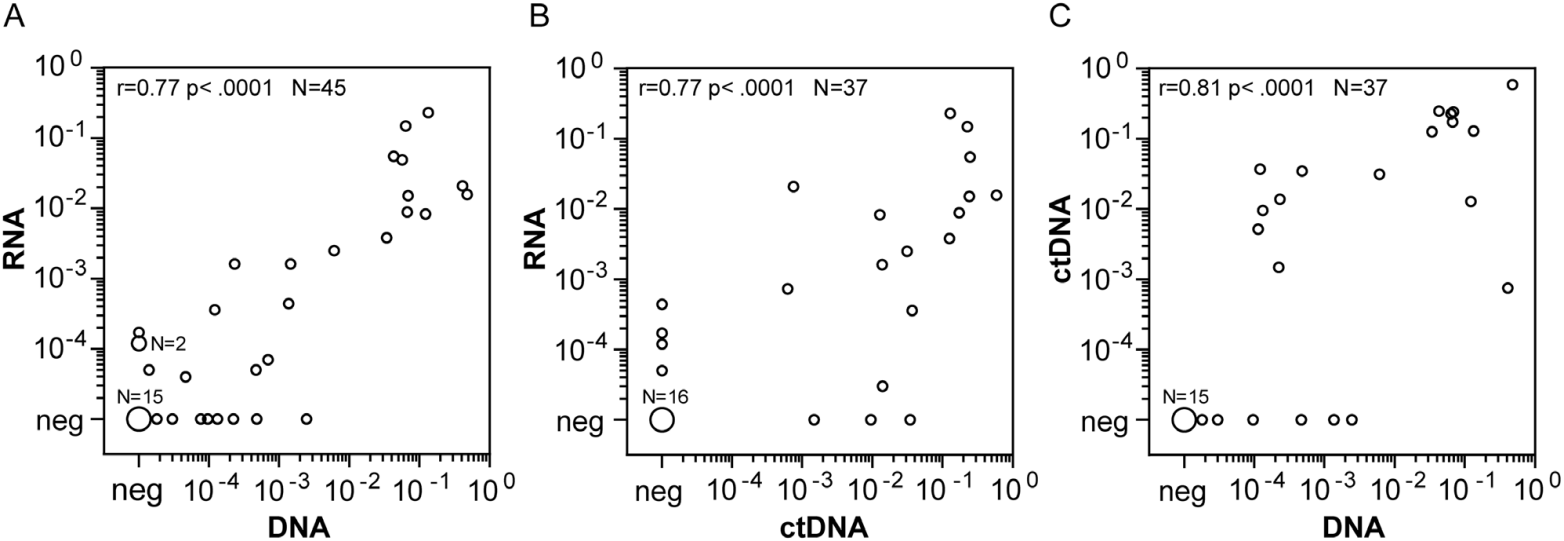
Comparison of quantitative *NPM-ALK* PCR results between cellular RNA, cellular DNA and cell free DNA Comparison of *NPM-ALK* copy numbers in blood or bone marrow samples from high risk ALCL-patients using the cellular RNA- and DNA-based and cell-free DNA-based methods. **(A)** Cell-based fusion transcripts (RNA) versus cellular fusion-sequence DNA-based (DNA), n=45. **(B)** Cell-based fusion transcripts (RNA) versus cell-free fusion-sequence DNA-based (ctDNA), n=37. **(C)** Cellular fusion-sequence DNA-based (DNA) versus cell-free fusion-sequence DNA-based (ctDNA), n=37.

The DNA breakpoint method and the standard RNA method were well correlated for *NPM-ALK* quantification, with a correlation coefficient of 0.77 (p < 0.0001) (Figure 3A). Eight samples identified as negative by the RNA-based method were identified as positive at the DNA level, usually with very low copy numbers (0.08, 0.2, 0.7, 0.8, 1, 2.1, 4.7, 24 *NPM-ALK*/10^4^ *ALB* copies). Three samples identified as negative at the DNA-level had measurable RNA-copies (1.1, 1.1, and 1.6 *NPM-ALK*/10^4^ *ABL* copies).

In 37 available concordant plasma and cell samples, we were able to perform *NPM-ALK* quantification with RNA in the cellular fraction and with cell-free circulating tumor DNA (ctDNA) in the plasma fraction. Spearman՛s correlation revealed a correlation of ctDNA quantification data with *NPM-ALK* RNA levels (r = 0.77, p < 0.0001) as well as with cellular *NPM-ALK* DNA levels (r = 0.81, p < 0.0001) (Figure 3).

**Figure 4:**
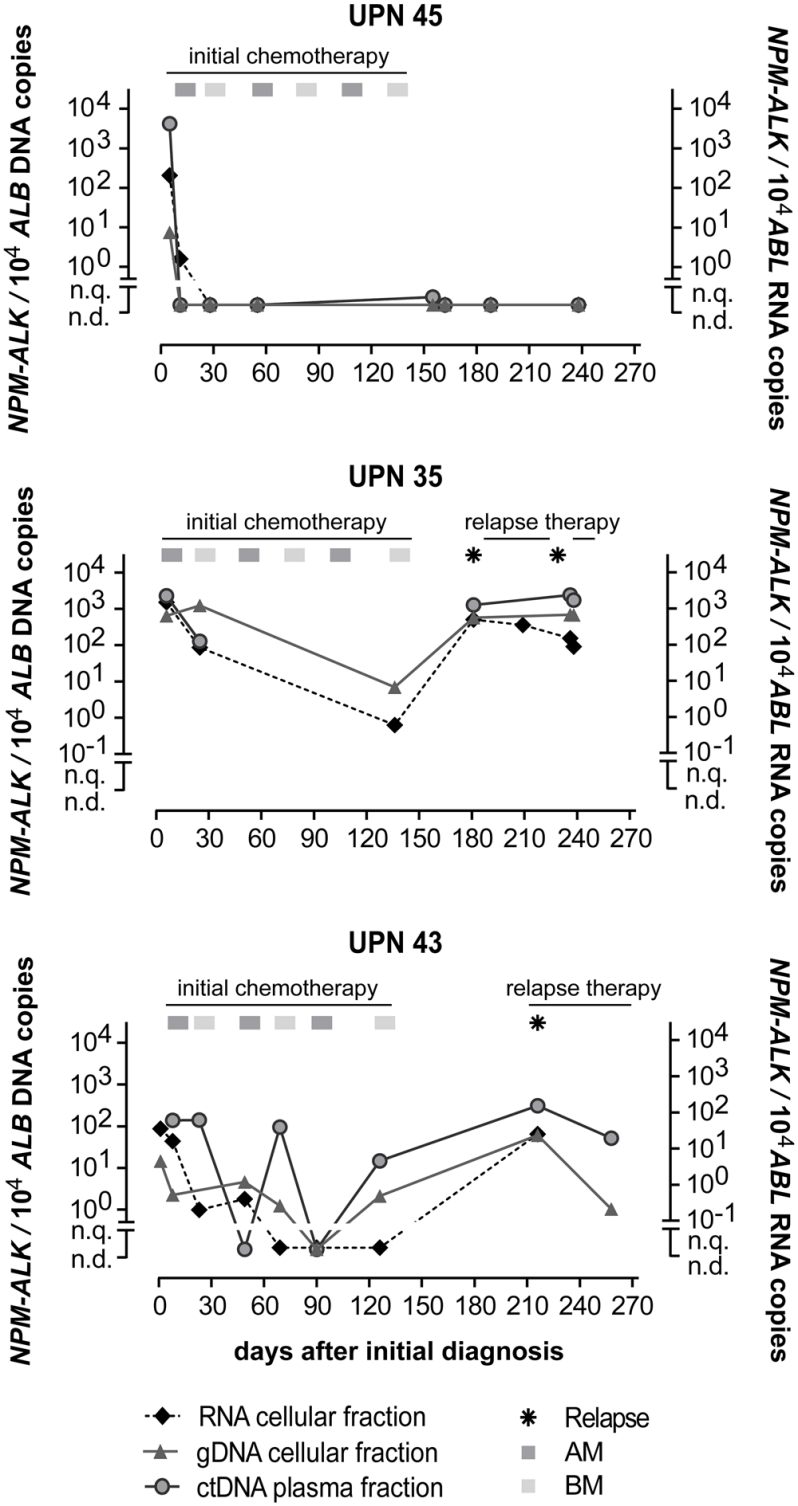
Quantification of *NPM-ALK* in ALCL patients *NPM-ALK* fusion RNA, DNA and ctDNA copies in 3 ALCL patients during their disease course. (AM… course of dexamethasone, methotrexate, ifosfamide, cytarabine and etoposide; BM… course of dexamethasone, methotrexate, cyclophosphamide and doxorubicine).

Examples of the course of MRD quantification of initially MDD-positive patients for three patients are shown in Figure 4. Patient UPN45 was MDD-positive using RNA and DNA based quantification. The patient’s MRD then became negative in cells (RNA and DNA level) and plasma (ctDNA) before the second course of chemotherapy, and stayed MRD negative in all available following timepoints according to all three methods. Patient UPN35, however, never became MRD negative according to any of the methods and suffered two relapses. These two patients showed complete concordance between all three methods.

Despite the overall correlation of the MRD results obtained by both the RNA and DNA methods, the MRD course of patient UPN43 contained two timepoints at which *NPM-ALK* was detectable by the DNA-based method at low copy numbers while fusion gene transcripts were not. The prognostic important MRD timepoint before the second course of chemotherapy [18], however, showed concordance in all three patients.

### *In vitro* evaluation of RNA- and DNA-based therapy monitoring during treatment with ALK kinase inhibitors

**Figure 5:**
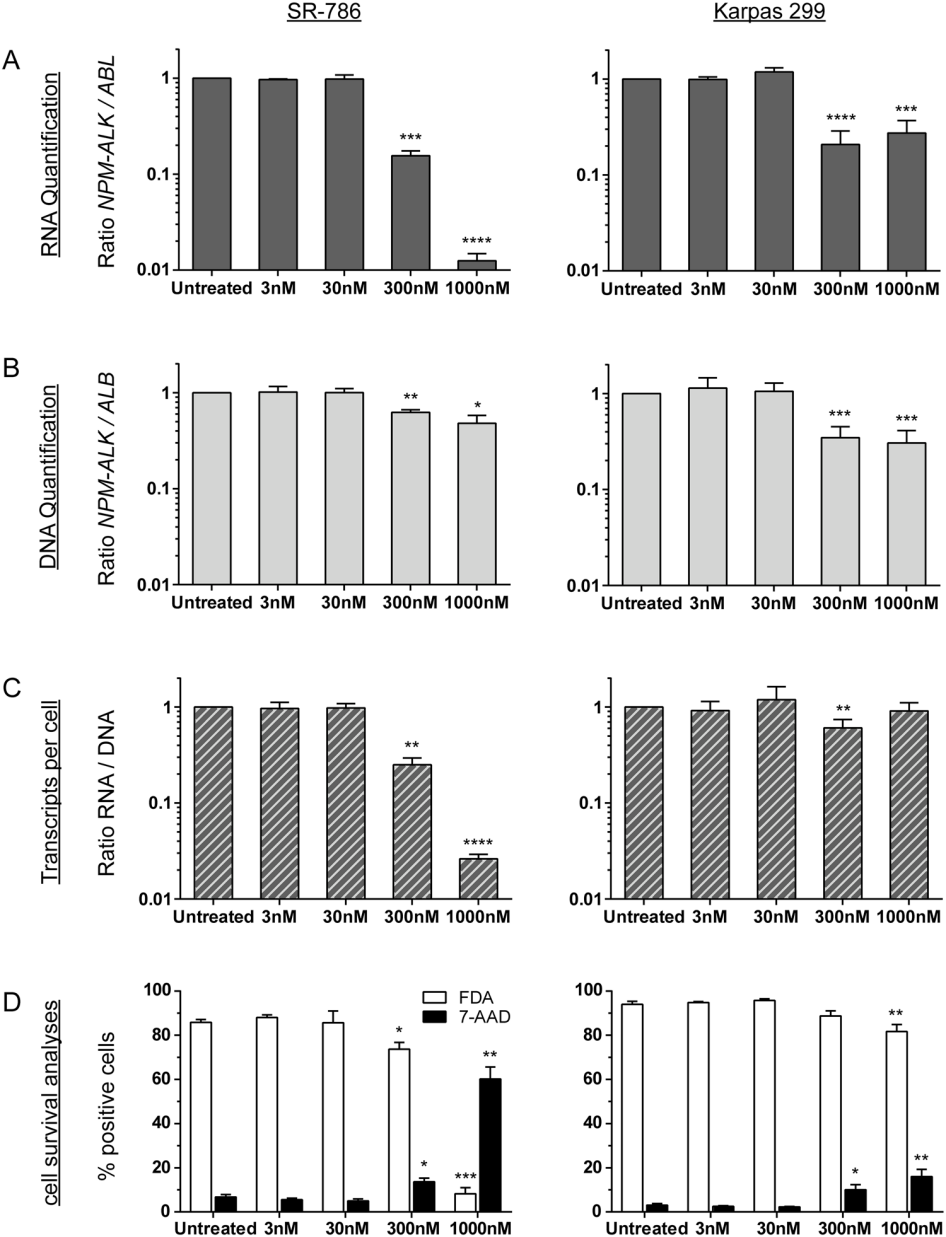
*NPM-ALK* quantification after crizotinib treatment *in vitro* Parallel quantification of *NPM-ALK* fusion transcript **(A)** and fusion gene **(B)** copies and cell survival analyses **(D)** after treatment of ALK-positive ALCL cell lines (SR-786 or Karpas 299) mixed 1:100 with an ALK-negative lymphoma cell line (DG75) with crizotinib for 72 h. Transcripts per cell were calculated based on quantified RNA and DNA copies **(C)**. Experiments were repeated three or five times for SR-786 and Karpas 299, respectively. (^****^ p < 0.0001, ^***^ p < 0.001, ^**^ p < 0.01, ^*^ p < 0.05).

To evaluate whether DNA-based minimal disease measurement might provide additional information beyond the standard RNA-based method, we compared RNA- and DNA-based quantification of *NPM-ALK* fusion sequences in two ALK+ ALCL cell lines (Karpas 299 and SR-786) incubated with different concentrations of the ALK kinase inhibitor crizotinib for 72 hours (Figure 5). To measure changes in *NPM-ALK* fusion genes and fusion gene transcripts under minimal disease conditions, ALK- negative DG-75 cells were added to the ALK+ ALCL cells for a dilution of 1:100. We also determined the amount of living and dead cells following the 72-hour exposure (Figure 5).

In the SR-786/DG75 cell suspension mix, we observed a concentration-dependent reduction of the *NPM-ALK* fusion transcripts after 72 hours of incubation with crizotinib (Figure 5A). At the DNA level, the reduction was far less pronounced (Figure 5B). The ratio of *NPM-ALK* RNA to DNA revealed reduced *NPM-ALK* expression per *NPM-ALK*-containing cell (Figure 5C). In concordance with the results of our cell death measurements, *NPM-ALK* quantification was not significant reduced at either the RNA level or the DNA level after 72 h of incubation with crizotinib. In contrast to SR-786 cells, the less sensitive cell line Karpas 299 (Figure 5D) shows a lower reduction of *NPM-ALK* RNA transcripts even after treatment with the highest concentration (1000 nM crizotinib) (Figure 5A) that results in nearly constant RNA/DNA ratios (Figure 5C).

## DISCUSSION

In the present study, we established a multiplex PCR assay for the detection of genomic *NPM-ALK* fusion sequences in children with ALK-positive ALCL in order to investigate the pattern of fusion sites in ALCL and to assess genomic breakpoints as biomarkers for minimal disease quantification. Our multiplex PCR assay identified the genomic fusion gene sequences in all 45 pediatric ALCL patients investigated, permitting further characterization of the breakpoint features and breakpoint distribution in a large cohort of ALK-positive pediatric ALCL patients.

Of the few genomic *NPM-ALK* fusion sequences that have been previously published, all were identified with long-range or nested long-range PCRs [6, 7, 23–25]. These methods require high-molecular-weight DNA that often cannot be retrieved from the formalin-fixed, paraffin-embedded tumor tissue available from routine diagnostics. Our multiplex PCR, however, is less dependent on high-quality DNA because it generates smaller amplification products. The *NPM-ALK* breakpoint distribution allows complete coverage of the breakpoint cluster regions with multiplex PCR compatible primer numbers. In principle, genomic fusion sequences can also be identified with library enrichment strategies and next generation sequencing techniques that allow parallel sequencing of several patients in an automated pipeline. Given the small breakpoint cluster regions in the *NPM and ALK* gene, easily accessible with one multiplex PCR assay, and the rare occurrence of this disease, costly enrichment assays in preparation for next generation sequencing may not offer substantial benefit for diagnostic laboratories.

The detailed sequence analysis of fusion site sequences revealed an NHEJ repair pattern similar to other chromosomal translocations in leukemia and sarcoma [26–31]. Features of VDJ recombination or AID signatures identified in B-cell lymphomas, Burkitt lymphoma, mantle cell lymphoma, or myeloma are not predominant in *NPM-ALK*-positive ALCL [21].

Detection and quantification of *NPM-ALK* fusion transcripts (MDD) as well as early MRD measurement have been established as independent prognostic markers in children and adolescents with ALK-positive ALCL [15–18]. We optimized the quantification of *NPM-ALK* fusion genes by choosing patient-specific primer and probe sets from the individual fusion sequences. Our results applying both RNA- and DNA-based minimal disease assessments on patient samples show that the patient-specific *NPM-ALK* DNA breakpoints can be used to design primers allowing for minimal disease assessment with at least the same sensitivity as the standard RNA-based method. Quantified copy numbers of the *NPM-ALK* fusion gene and *NPM-ALK* fusion transcript correlate well. The results obtained by the RNA-based method have proven prognostic value for patients with ALCL. In addition, the method does not require identification of the DNA-breakpoint and development of a patient-specific assay. Therefore the assessment of MDD and MRD on the RNA-level is the standard method during current chemotherapy. In addition, several examples show that MRD-analysis for *NPM-ALK* transcripts is helpful for guiding treatment decisions in patients with very high risk or relapsed ALCL [32, 33]. New treatment options against ALCL like ALK-kinase inhibitors are arising. These agents induce cell cycle arrest in *in vitro* cell line experiments with variable effects on cell death [34, 35]. Clinically, rapid development of relapses with MRD-reappearance has been observed after discontinuation of crizotinib in patients treated with the drug for ALCL-relapse, suggesting that quiescent tumor cells that are detectable by DNA based methods, but are underestimated by RNA-based methods might exist [33]. We therefore suggest studying possible clinical implications of DNA-based MRD-screening in addition to the standard method in clinical trials with ALK-inhibitors.

The high stability of DNA furthermore enables the quantification of cell-free tumor DNA (ctDNA) that is released into the plasma from primary tumor and metastasis [36–38]. For many solid cancers, *e.g.,* colorectal, lung, and breast cancer, or Ewing sarcoma, quantification of ctDNA has been established as a valuable tool for non-invasive therapy monitoring and even risk stratification [38–41].

Mussolin *et al.* investigated the presence of total cell-free DNA and *NPM-ALK* fusion sequences in initial plasma samples of 43 NPM-ALK-positive ALCL patients [42]. They used a SYBR green-based real-time PCR assay and the same primer pair for *NPM-ALK* quantification for all patients. They observed no correlation of the total cell-free DNA with the presence or absence of MDD as determined by qualitative PCR for *NPM-ALK* transcripts in mononuclear cells. In addition, their method did not analyze whether there was any association between the amount of cell-free *NPM-ALK* DNA and the presence or absence of MDD. In our patient-specific assays, genomic *NPM-ALK* copies in the patient՛s plasma correlated with both the amount of *NPM-ALK* DNA and RNA fusion sequences in the cellular blood fraction, suggesting the release of tumor DNA from circulating cells or a sign of total tumor burden. The correlation of ctDNA with classical MDD/MRD quantification as well as the concordance of all results in samples obtained during therapy suggests a possible prognostic role for quantification ctDNA in patients with ALK-positive ALCL. However, that possibility will need to be further analyzed in a larger, unselected patient cohort.

In summary, we established a multiplex PCR assay for reliable identification of ALCL patients’ individual genomic *NPM-ALK* fusion sequences that can be easily adopted for routine diagnostics and enables a DNA-based minimal disease monitoring for ALK-positive ALCL patients. We propose that supplementary MRD assessment including RNA and DNA quantification may allow for better understanding the mode of action of new targeted therapies and may contribute to improved therapy assessment and risk stratification by detecting quiescent tumor cells.

## MATERIALS AND METHODS

### Patients and material

Cryopreserved tumor material and EDTA-blood or -bone marrow from NPM-ALK-positive ALCL patients included in the Berlin-Frankfurt-Muenster group study NHL-BFM95 or the NHL-BFM Registry 2012, or German patients enrolled in the European intergroup trial ALCL99 was included in the analysis after informed consent of the patients or their legal guardians. Both the studies and the registry were approved by the institutional ethics committee of the primary investigator of the NHL-BFM study group (A.R., W.W.). Tumor samples were available from 45 patients. The tumor material was from the initial biopsy in 25 patients and from a relapse biopsy in 15 patients, and the bone marrow or peripheral blood from five patients with high amount of circulating tumor cells measured by *NPM-ALK*-specific quantitative real-time PCR. Patient characteristics are shown in Table 1. The patient cohort was not representative with overrepresentation of relapse patients since tumor cells for sequencing was from relapse in several patients and infiltrated blood/bone marrow was used as well.

### Nested multiplex PCR assay for identification of genomic *NPM-ALK* fusion sites in ALCL patients

The genomic *NPM-ALK* fusion sequence was analyzed in four ALCL cell lines (Karpas 299, SR-786, L-82, and SuDHL-1) and 45 ALCL patients. Genomic DNA was isolated from tumor samples, bone marrow, or blood samples by Trizol reagent (Thermo Fisher Scientific).

To amplify genomic *NPM-ALK* fusion sequences, we developed a two-round multiplex PCR assay. For the first round, 100 ng of DNA was combined with one forward primer located at the 5՛ end of the *NPM* breakpoint cluster region (∼1kb; exons 4–5 [Chr5:170,818,710-170,819,820]) and five reverse primers covering the *ALK* breakpoint cluster region (∼2.2kb; exons 19–20 [Chr2:29,448,431-29,446,208]) to enable targeted amplification of PCR products with a maximal length of several hundred base pairs. Primer sequences are shown in Supplementary Table 2. Next, the amplified DNA was used in second-round single PCRs with corresponding nested primers. In five separate PCR reactions, the internal *NPM* forward primer was combined with one of the five internal *ALK* reverse primers to identify the *ALK* primer located closest to the fusion site. Systematic optimization of multiplex PCR parameters was carried out with DNA from *NPM-ALK*-positive cell lines L-82 and SR-786. To calculate the sensitivity of the multiplex PCR assay, we quantified a dilution series of *NPM-ALK*-positive cells with *NPM-ALK* negative HL60 cells were quantified. A minimum of one tumor cell in thousand wild-type cells can be detected by the multiplex PCR assay.

For identification of *ALK* fusion sequences with less common fusion partner genes *ATIC* and *TPM3* analogous nested multiplex PCR assays were established with one forward primer covering the breakpoint cluster region of *ATIC* (∼5.8 kb; exons 7–8 [Chr2:216,191,545-216,197,230]) and two forward primers covering the breakpoint cluster region of *TPM3* (∼13 kb; exons 6-8 [Chr1:154,143,187-154,130,198]).

The amplification product was sequenced after purification with the QIAquick PCR Purification Kit (Qiagen). Patient-specific breakpoints were confirmed in an independent PCR using primer sets next to the patient’s fusion site and 50 ng original tumor DNA. All PCR reactions were performed with the LongAmp^®^ Taq DNA Polymerase System (NEB) according to the manufacturer's instructions.

### Analysis of breakpoint characteristics

Patient-specific *NPM-ALK* fusion sequences were aligned to the human genome (hg19, UCSC Genome Browser). Breakpoint positions are listed in Table 1. Repeat elements at the fusion sites were identified with the RepeatMasker tool (http://www.repeatmasker.org/). Genomic fusion sites were then analyzed for co-localization with repeat elements, recombination-related DNA sequence motifs, Topoisomerase II binding sites, Translin binding sites, heptamer/nonamer recombination signals, recombination signal sequences (Recombination Signal Sequences Site tool, http://www.itb.cnr.it/rss/), palindromic sequences (EMBOSS explorer http://emboss.bioinformatics.nl/cgi-bin/emboss/palindrome and Palindromic sequences finder tool http://www.biophp.org/minitools/find_palindromes/demo.php), human minisatellites core sequence, human minisatellites conserved sequence, hypervariable minisatellites recombination sequence, DNA polymerase frameshift hotspots, immunoglobulin heavy chain class switch repeats, LTR-IS motifs, and human replication origin consensus sequence.

Components of the free software environment R (http://www.r-project.org) were used for kernel density analysis as described previously [30].

### Quantification of tumor specific RNA, genomic DNA, and cell-free circulating DNA using the individual *NPM-ALK* fusion sequence

As a proof of principle, eight high-risk patients with detectable *NPM-ALK* fusion transcripts were monitored during treatment course by parallel quantification of the *NPM-ALK* fusion transcript in blood or bone marrow cells, the *NPM-ALK* fusion gene in blood or bone marrow cells and the *NPM-ALK* fusion gene in cell-free plasma samples. In total, 50 RNA samples, 48 DNA samples and 42 plasma samples were analyzed. Genomic DNA and RNA were isolated from bone marrow or peripheral blood samples using Trizol reagent (Thermo Fisher Scientific). cDNA synthesis was performed using 1 μg total RNA, random hexamers, and superscript II reverse transcriptase (Invitrogen). Cell-free circulating DNA was isolated from frozen plasma samples with the QIAamp Circulating Nucleic Acid Kit (Qiagen).

For MRD monitoring, the *NPM-ALK* fusion transcripts (RNA) were quantified using real-time quantitative PCR as previously described [17]. Genomic *NPM-ALK* fusion sequences (DNA) were quantified with digital droplet PCR QX200 Reader (BioRad) using patient individual breakpoint spanning primers and probe sets (Supplementary Table 3). To calculate the absolute number of *NPM-ALK* copies, the fusion-specific probe signal was normalized to a signal of the single-copy human albumin gene.

### Comparative analysis of *NPM-ALK* fusion transcript and fusion gene levels after treatment of ALK+ ALCL cell lines with ALK kinase inhibitor

*NPM-ALK*-positive (ALK+) cell lines Karpas 299 (a cell line with lower sensitivity to the ALK kinase inhibitor crizotinib) and SR-786 (a cell line with higher sensitivity to the ALK kinase inhibitor crizotinib) and *NPM-ALK* negative (ALK-) cell line DG-75 were obtained from the German Resource Centre for Biologic Material (DSMZ) and were cultured in RPMI medium supplemented with 10% fetal bovine serum, L-glutamine, and antibiotics at 37°C in 5% CO_2_. The ALK kinase inhibitor crizotinib was obtained from Cell Signaling Technology. Stock solutions (10 mM) were prepared with DMSO and stored at -80°C; working solutions were prepared with DMSO immediately before use.

50,000 ALK+ cells were incubated with increasing concentrations (3 nM, 30 nM, 300 nM and 1000 nM, respectively) of crizotinib for 72 h. To measure changes of *NPM-ALK* fusion genes and fusion gene transcripts under MRD conditions, 4,950,000 ALK- DG-75 cells were added for a dilution of 1:100.

For quantification of *NPM-ALK* fusion transcripts and fusion genes, RNA and DNA were isolated in parallel using the AllPrep DNA/RNA Mini Kit (Qiagen). cDNA synthesis was performed with 1 μg of RNA, random hexamer primers, and Superscript II reverse transcriptase (Invitrogen). *NPM-ALK* transcripts and fusion genes were quantified using fusion-sequence-spanning primers and probes (Supplementary Table 3). The *NPM-ALK* fusion transcript was normalized to the housekeeping gene *ABL1* to exclude experimental variation during the cDNA synthesis process. To calculate the absolute number of ALK+ cells, the *NPM-ALK* fusion gene signal was normalized to a signal of the single copy gene albumin, which is equally detectable in ALK+ and ALK- cells.

In addition, 100,000 ALK+ (Karpas 299 and SR-786) and ALK- cells (DG-75) were analyzed in two parallel experiments to assess the number of living and dead cells after 72 h of crizotinib treatment. To detect viable cells, the cell lines were incubated with 5 ng/ml fluorescein diacetate (FDA) (Sigma) for 20 minutes at 37°C. Cells were collected by centrifugation and re-dissolved in 200 μl PBS. To detect dead cells, 10 μl 7-AAD solution (BD-biosciences) was added and the cells were stained on ice for 20 minutes [43]. The number of viable and dead cells was measured on a FACS Calibur flow cytometer with Cell Quest Pro software (BD biosciences). Analysis was performed with FlowJo 10 software (Miltenyi Biotech).

### Statistical analysis

Co-localization of genomic breakpoints to repeat regions and DNA sequence motifs was statistically analyzed using the Fisher՛s exact test. Differences between mean values of the *in vitro* measurements were assessed with a one-way ANOVA test. MDD data from the quantification of the *NPM-ALK* fusion at the RNA, DNA, and ctDNA levels were compared using Spearman correlation statistics.

## SUPPLEMENTARY MATERIALS TABLES





## Abbreviations

ALCL: anaplastic large-cell lymphoma
MDD: minimal disseminated disease
MRD: minimal residual disease
*NPM-ALK*: *Nucleophosmin–anaplastic lymphoma kinase*
