# Supplementary material for: Characterization and diagnostic application of genomic NPM-ALK fusion sequences in anaplastic large-cell lymphoma
Source: Oncotarget. 2018 May 29;9(41):26543–55. doi: 10.18632/oncotarget.25489 (PMC5995187; doi:10.18632/oncotarget.25489)
Supplement: Supplementary file 1 [file oncotarget-09-26543-s001.pdf]

Characterization and diagnostic application of genomic *NPM-ALK* fusion sequences in anaplastic large-cell lymphoma

SUPPLEMENTARY MATERIALS

Supplementary Table 1: Monitoring of *NPM-ALK* fusion RNA, DNA and ctDNA levels in 8 ALCL patients during the treatment course

See Supplementary File 1

Supplementary Table 2: Sequences of primers used in nested multiplex PCR assay

		Primer	Sequence 5´-3´
NPM	external	NPM_fw_1	acatgggggcttctgctgctacttttatc
	internal	NPM_fw_2	atcagaggtggaaaaacaggttcactgg
ATIC	external	ATIC_fw_1	agccaccacatagcactgaatagtgaggag
	internal	ATIC_fw_2	tttgctgtggacgtagaaaaccatcttgtc
TPM3	external	TPM3_fw_1	tggaccattcagcacatttagtgcaagag
	internal	TPM3_fw_2	gggggtattgcctgtgtgactaatttctac
	external	TPM3_fw_3	ttggagggaaagaagacggttgagtagttc
	internal	TPM3_fw_4	tggatgctttctagtggtttttcaacagag
ALK	external	ALK_rv_1	gttctggtggttgaatttgctgcagag
	internal	ALK_rv_2	tttgctgcagagcagagagggatgtaac
	external	ALK_rv_3	gaggaggtcttgccagcaaagcagtag
	internal	ALK_rv_4	agttggggttgtagtcggtcatgatgg
	external	ALK_rv_5	tagggcagagtcatgttagtctggttcctc
	internal	ALK_rv_6	gaagcagactggagatgggattagacc
	external	ALK_rv_7	catgtgaggatgttctggaaggcaaactc
	internal	ALK_rv_8	gaaggcaaactccatggaagccagaac
	external	ALK_rv_9	ccttggctcacaggctgaacagaaatatac
	internal	ALK_rv_10	aaccgattttcctatctctctgcctggag

Supplementary Table 3: Sequences of primers and probes used in ddPCR for quantification of the genomic *NPM-ALK* fusion sequence

**UPN12**	forward primer	5´-AAGTCTCAGTTTTTAGAGTATTTACTATCAGTG-3´
	probe	5´-ATTGGTGAGTGCACAGAGCCCCAG-3´
	reverse primer	5´-ACTATTCAGTCCTGCCTTCCTG-3´
**UPN15**	forward primer	5´-CTCATTATAACGGCTGGAGTGC-3´
	probe	5´-CCCGTGTGTATCCTCCTGGCTGATC-3´
	reverse primer	5´-TGTGGGAGCTAGAAGTGACG-3´
**UPN26**	forward primer	5´-AAGTCTCAGTTTTTAGAGTATTTACTATCAGTG-3´
	probe	5´-TTCTTTTCCCAACCTTCCCCCCAC-3´
	reverse primer	5´-GCTGAGGTGCAGAATCAGG-3´
**UPN35**	forward primer	5´-CTCATTATAACGGCTGGAGTGC-3´
	probe	5´-CATAGCTTGCTATATCGAAAATCTGCAGACCATATTG-3´
	reverse primer	5´-GGCAGAGTCATGTTAGTCTGGTTC-3´
**UPN41**	forward primer	5´-CTCATTATAACGGCTGGAGTGC-3´
	probe	5´-ACTCAGAAACCGATTATGATCACATCACTGCA-3´
	reverse primer	5´-TCAGCTCACCTTGGCTCAC-3´
**UPN43**	forward primer	5´-TGTATATGTGCAGATGGGGTCTC-3´
	probe	5´-ATGTTGCCCAGATTGGTGCCATCCCT-3´
	reverse primer	5´-GCACGTAGTAACCATGCAACAAG-3´
**UPN44**	forward primer	5´-GCTTGGTTCCCAGTTTGGAC-3´
	probe	5´-CTCTCAAGAGCCTTTCCCGTACTATACCCATG-3´
	reverse primer	5´-TCTACCGGCAGATCCCTTTG-3´
**UPN45**	forward primer	5´-CTTGCTGCTTGAGTTTTATAATGTC-3´
	probe	5´-TTAAAGAAGGTGTGTCTTTTCCTCCACAGCTAAAA-3´
	reverse primer	5´-AGCTCTGAACCTTTCCATCATAC-3´
**Karpas 299**	forward primer	5´-gcctccccaaagtgagattac-3´
	probe	5´-cctccccggccAAGCCAAAAC-3´
	reverse primer	5´-GCTGAGGTGCAGAATCAGG-3´
**SR-786**	forward primer	5´-GAACTCCTGGCCTTAACGTG-3´
	probe	5´-ACGCCCTGGCGGCTCCAGAA-3´
	reverse primer	5´-GAAGGTTGGGTGGAAGCAC-3´
**human albumin control**	forward primer	5´-TGAAACATACGTTCCCAAAGAGTTT-3´
	probe	5´-TGCTGAAACATTCACCTTCCATGCAGA-3´
	reverse primer	5´-CTCTCCTTCTCAGAAAGTGTGCATAT-3´
