# Supplementary material for: Characterization and diagnostic application of genomic NPM-ALK fusion sequences in anaplastic large-cell lymphoma
Source: Oncotarget. 2018 May 29;9(41):26543–55. doi: 10.18632/oncotarget.25489 (PMC5995187; doi:10.18632/oncotarget.25489)
Supplement: Supplementary file 2 [file oncotarget-09-26543-s002.docx]

**Supplemental Table 1:**

Monitoring of *NPM-ALK* fusion RNA, DNA and ctDNA levels in 8 ALCL patients during the treatment course.

**UPN12**

| **month after diagnosis** | 37* | 38 | 50 | 63 | 69 | 76 | 114* | 120 | 123 | 125 |
| --- | --- | --- | --- | --- | --- | --- | --- | --- | --- | --- |
| **RNA** (*NPM-ALK /* 10^4^ *ABL* copies) | 3.50 | n.d. | n.d. | n.d. | n.d. | 1.10 | n.d. | n.d. | 1.10 | n.d. |
| **DNA** (*NPM-ALK /* 10^4^ *ALB* copies) | 1.11 | n.d. | n.d. | n.d. | 0.20 | n.d. | n.d. | 0.08 | n.d. | n.d. |
| **ctDNA** (*NPM-ALK /* 10^4^ *ALB* copies) | 370.91 | - | n.d. | - | n.d. | - | n.d. | n.d. | n.d. | - |

**UPN15**

| **month after diagnosis** | 26* | 43 | 45 | 75 |
| --- | --- | --- | --- | --- |
| **RNA** (*NPM-ALK /* 10^4^ *ABL* copies) | 550.00 | 0.30 | n.d. | n.q. |
| **DNA** (*NPM-ALK /* 10^4^ *ALB* copies) | 431.27 | 0.37 | 0.66 | n.d. |
| **ctDNA** (*NPM-ALK /* 10^4^ *ALB* copies) | 2463.94 | - | - | n.d. |

**UPN26**

| **month after diagnosis** | 1 | 12* | 35* | 40 | 58 | 66 |
| --- | --- | --- | --- | --- | --- | --- |
| **RNA** (*NPM-ALK /* 10^4^ *ABL* copies) | n.d. | n.d. | - | 0.40 | n.d. | n.d. |
| **DNA** (*NPM-ALK /* 10^4^ *ALB* copies) | n.d. | 4.71 | - | 0.04 | n.d. | n.d. |
| **ctDNA** (*NPM-ALK /* 10^4^ *ALB* copies) | n.d. | 346.72 | - | n.q. | n.d. | n.d. |

**UPN35**

| **days after diagnosis** | 6 | 25 | 136 | 181* | 209 | 229* | 236 | 238 |
| --- | --- | --- | --- | --- | --- | --- | --- | --- |
| **RNA** (*NPM-ALK /* 10^4^ *ABL* copies) | 1487.00 | 83.00 | 0.60 | 490.00 | 350.00 | - | 151.00 | 88.00 |
| **DNA** (*NPM-ALK /* 10^4^ *ALB* copies) | 637.83 | 1226.19 | 6.90 | 570.18 | - | - | 691.16 | 668.69 |
| **ctDNA** (*NPM-ALK /* 10^4^ *ALB* copies) | 2261.73 | 127.45 | n.q. | - | - | - | 2407.41 | 1733.33 |

**UPN41**

| **month after diagnosis** | 5* | 6 |
| --- | --- | --- |
| **RNA** (*NPM-ALK /* 10^4^ *ABL* copies) | 2283.00 | 156.00 |
| **DNA** (*NPM-ALK /* 10^4^ *ALB* copies) | 1333.33 | 4787.30 |
| **ctDNA** (*NPM-ALK /* 10^4^ *ALB* copies) | 1282.05 | 5882.35 |

**UPN43**

| **days after diagnosis** | 1 | 8 | 23 | 49 | 69 | 90 | 126 | 216* | 258 |
| --- | --- | --- | --- | --- | --- | --- | --- | --- | --- |
| **RNA** (*NPM-ALK /* 10^4^ *ABL* copies) | 16.00 | 16.00 | 0.20 | 0.40 | n.d. | n.d. | n.d. | 25.00 | - |
| **DNA** (*NPM-ALK /* 10^4^ *ALB* copies) | 14.58 | 2.24 | - | 4.58 | 1.23 | n.d. | 2.12 | 60.49 | 1.04 |
| **ctDNA** (*NPM-ALK /* 10^4^ *ALB* copies) | - | 138.44 | 140.35 | n.d. | 95.24 | n.d. | 14.65 | 310.88 | 51.34 |

**UPN44**

| **days after diagnosis** | 162* | 181 | 186 | 209 | 214 | 256 |
| --- | --- | --- | --- | --- | --- | --- |
| **RNA** (*NPM-ALK /* 10^4^ *ABL* copies) | 38.00 | 4.30 | 7.20 | n.d. | n.d. | n.d. |
| **DNA** (*NPM-ALK /* 10^4^ *ALB* copies) | 342.74 | 13.56 | - | 24.45 | 0.86 | n.d. |
| **ctDNA** (*NPM-ALK /* 10^4^ *ALB* copies) | 1258.00 | n.d. | 6.12 | n.d. | n.d. | n.d. |

**UPN45**

| **days after diagnosis** | 5 | 11 | 28 | 55 | 155 | 162 | 188 | 238 |
| --- | --- | --- | --- | --- | --- | --- | --- | --- |
| **RNA** (*NPM-ALK /* 10^4^ *ABL* copies) | 207.00 | 1.60 | n.d. | n.d. | n.q. | n.d. | n.d. | n.d. |
| **DNA** (*NPM-ALK /* 10^4^ *ALB* copies) | 4130.89 | n.d. | n.d. | n.d. | n.q. | n.d. | n.d. | n.d. |
| **ctDNA** (*NPM-ALK /* 10^4^ *ALB* copies) | 7.46 | n.d. | n.d. | n.d. | n.d. | n.d. | n.d. | n.d. |

* relapse

n.q. non quantifiable

n.d. non detectable
